# Leave it alone: the natural history of growth-friendly graduates without a final fusion

**DOI:** 10.1007/s43390-025-01187-9

**Published:** 2026-01-24

**Authors:** Rayyan Abid, Bryan O. Ren, Robert F. Murphy, Jeffrey R. Sawyer, John M. Flynn, John B. Emans, John T. Smith, Paul D. Sponseller, Norman Ramirez, Christina K. Hardesty, Pediatric Spine Study Group

**Affiliations:** 1 Department of Orthopaedic Surgery, University Hospitals Rainbow Babies and Children’s, 2101 Adelbert Rd, Cleveland, OH 44106 USA; 2 School of Medicine, Case Western Reserve University, Cleveland, OH USA; 3 Department of Orthopaedic Surgery, University of Michigan, Ann Arbor, MI USA; 4 Medical University of South Carolina/Charleston VA, Charleston, SC USA; 5 Campbell Clinic Orthopaedics, Memphis, TN USA; 6 The Children’s Hospital of Philadelphia, Philadelphia, PA USA; 7 Boston Children’s Hospital, Boston, MA USA; 8 Primary Children’s Hospital, Salt Lake City, UT USA; 9 Johns Hopkins University, Baltimore, MD USA; 10 La Concepcion Hospital, San German, PR USA; 11 Pediatric Spine Foundation, Valley Forge, PA USA

**Keywords:** Early onset scoliosis, Graduate, Implants, Complications, UPROR

## Abstract

**Introduction:**

The natural history of growth-friendly graduates treated with growing instrumentation but no final fusion is unknown. Two previous reports exist with one analyzing 30 growin g rod patients with no definitive fusion and another including 10 patients with growing rod removal, but no comprehensive data exist in the literature.

**Methods:**

A multi-center database was queried for patients treated with TGR or VEPTR and at least 2 years of follow-up from their index procedure. Patients met inclusion criteria if they had not undergone a final fusion procedure but had completed planned interventions for early onset scoliosis and had sufficient follow-up. Kaplan–Meier analysis was performed to model rates of UPROR and complications in patients with retained and removed implants over time.

**Results:**

Of 233 included patients, definitive treatment was implant maintenance in 203 (87%) and removal in 30 (13%). Patients with retained implants experienced 10 (5%) UPRORs and 41 (20%) complications after final lengthening. Patients with removed implants had 0 UPRORs and 10 (33%) complications after implant removal. Patients with available pre-definitive and follow-up measurements and whose implants were removed increased by a mean 14.3° of coronal curvature compared to 19.0° in those who retained implants at mean 3.8 and 3.7 year follow-up, respectively.

**Conclusion:**

Growth-friendly graduates without final fusion demonstrate low rates of UPROR and complications following definitive lengthening or implant removal. However, those who undergo implant removal are more likely to experience complications. Coronal curve magnitude was moderately maintained in both cohorts, suggesting that avoidance of definitive fusion may be viable for some patients.

**Supplementary Information:**

The online version contains supplementary material available at 10.1007/s43390-025-01187-9.

## Introduction

Early onset scoliosis (EOS) is defined as spinal deformity occurring in children 10 years of age and younger [1]. Scoliosis is often treated with definitive fusion to prevent further curve progression, but such fusion in very young patients has been linked to a number of complications, including thoracic growth arrest and associated pulmonary issues [2–4]. Thus, there has been a significant increase in management of spinal deformity in immature children with growing instrumentation in recent years [5]. These adjustable techniques attempt to maximize thoracic growth while limiting the risk of thoracic insufficiency [6]. Typically, implants used for this purpose are used to delay further procedures and are not intended to be left in situ definitively, but some circumstances may prevent their removal. In other cases, growing instrumentation may be retained by choice and no definitive fusion procedure is performed. Finally, patients may have these implants removed but still elect to forego definitive fusion.

Currently, there exists limited information regarding outcomes of patients who have completed their growing rod programs, termed ‘growing rod graduates,’ and choose to forego definitive spinal fusion [7]. Previous reports have evaluated subgroups of patients with growing rod constructs who did not undergo a definitive fusion and compared them to those with definitive fusion, but there are no studies that have directly compared patients with retained and removed implants without definitive fusion in a cohort of this size [8, 9]. Thus, the aim of this study was to evaluate these outcomes in a large, multi-center data set, analyzing the natural history of children who underwent either TGR or VEPTR constructs but did not undergo a “final fusion” procedure. We also wished to compare rates of UPROR and complications in those who retained versus removed their implants. Complications refer to any adverse outcome recorded for each patient whereas UPROR refers specifically to complications that necessitated operative treatment. Our hypotheses are that the natural history of this cohort is uneventful, that curve magnitudes are better maintained in patients who retain their implants compared to those whose implants are removed, and that unplanned return to the operating room (UPROR) rates are similar in these groups.

## Methods

**Fig. 1:**
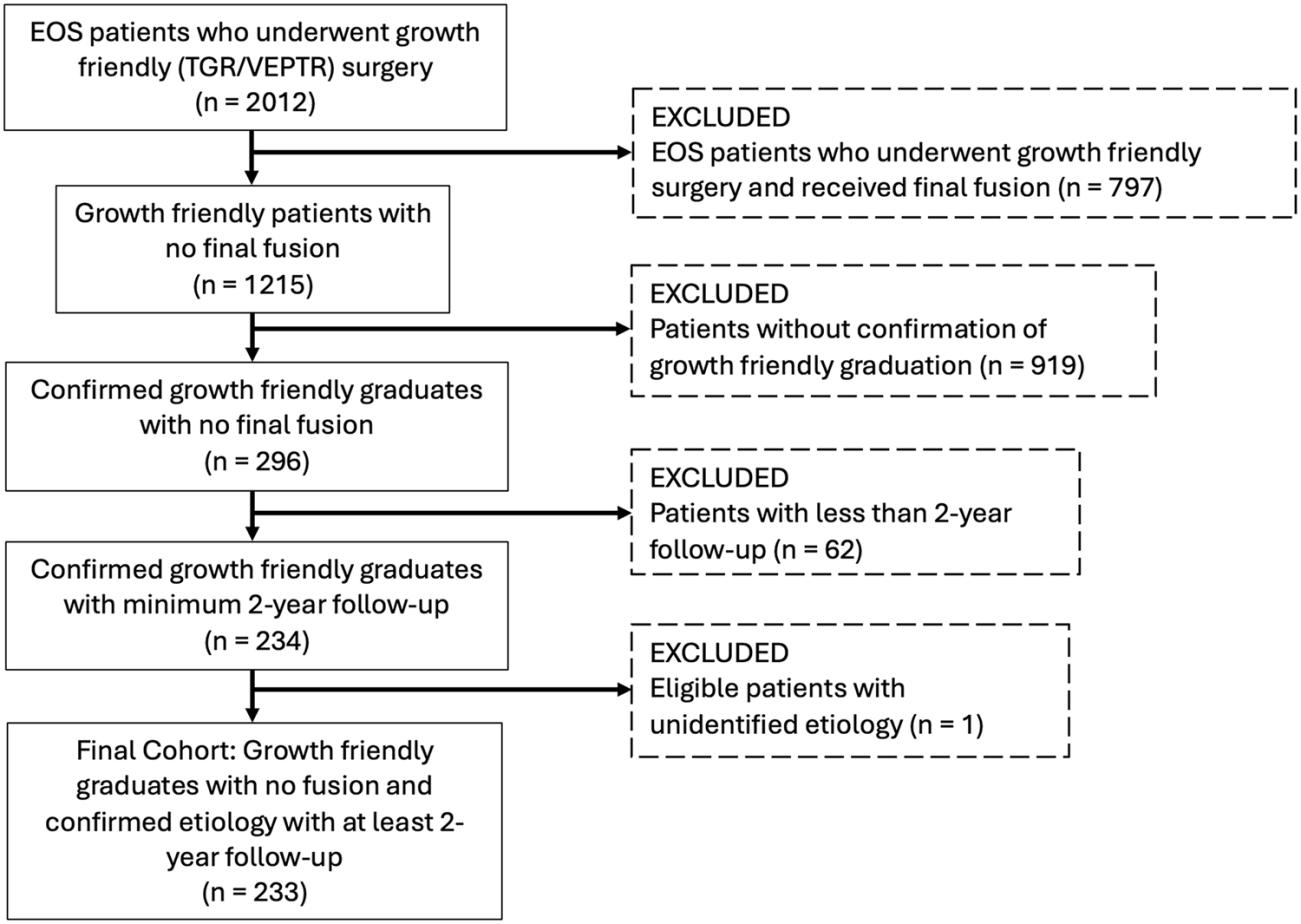
STROBE diagram depicting patient selection process

The Pediatric Spine Study Group database was queried for patients with TGR or VEPTR constructs who had at least 2 years of follow-up after their final intervention. In all cases, the surgeon decided and/or shared decision-making with the family led to stop lengthening, but not to perform a definitive fusion. In our initial query, we identified 1215 patients who had either TGR or VEPTR implanted but had not yet had a definitive fusion. Of those, 296 had their parents complete the STOP questionnaire and were confirmed as “graduates,” indicating there were no plans for additional lengthening or fusion procedures. Of the “graduates,” 62 (20.9%) patients excluded due to insufficient follow-up and 1 was excluded due to unidentified etiology, leaving 233 (78.7%) patients in the final cohort (Fig. 1). Demographic data included age, gender, race, etiology, diagnosis, comorbidities, height, weight, ambulatory status, and prior treatment. Radiographic data included major/minor Cobb angles and levels, spinal height, sagittal kyphosis, and proximal junctional kyphosis.

In addition to descriptive statistics regarding relative rates of UPROR and complications for each group, Kaplan–Meier survival analysis was performed to predict the probability of each outcome for both those who retained and removed their implants over time. Changes in coronal curve magnitude following definitive procedure were compared for patients who had these measurements available. Mann–Whitney U test was performed to determine if these changes were significantly different between both groups. For each group, we also performed a linear regression comparing coronal curvature following patients’ definitive procedures and curve progression at latest follow-up.

We also analyzed cohorts’ baseline characteristics to determine whether or not they were similar. After categorizing groups into those with and without comorbidities, Fisher’s exact test was performed to compare relative rates of comorbidities. Fisher’s exact test was also performed to compare etiologies. A two-sample *t* test was also used to determine whether pre-index coronal curvature was similar between groups. *P* values less than 0.05 were considered significant. Statistical analysis was performed using R software (version 4.4.1 for MacOS).

## Results

The sample exhibited a heterogenous collection of diagnoses including 99 (42.5%) patients with congenital scoliosis, 71 (30.5%) with neuromuscular scoliosis, 43 (18.5%) with syndromic scoliosis, and 20 (8.6%) with idiopathic scoliosis. Mean age at the time of definitive procedure (either final lengthening or growth construct removal) was 12.7 ± 2.6 years old (3.8–21.0 years). One hundred six (45.5%) patients were male and one hundred twenty-seven (54.5%) were female. The mean time between patients’ definitive surgery date and latest follow-up date was 4.1 ± 2.4 years (0.3–18.8 years). The mean time between patients’ index procedure and latest follow-up date was 10.1 ± 3.4 years (2.8–20.9 years). At the completion of lengthening procedures, 203 of the 233 patients (87.1%) had no additional surgeries and their implants were retained. Implants were removed in 30 of the 233 patients (12.9%). Within this cohort, 11 (36.7%) patients removed implants due to some complication, including device migration, infection, hardware prominence, hardware failure, and junctional kyphosis. The remaining 19 (63.3%) removed implants as a planned step in their treatment.

Mean age at the time of definitive procedure was 12.8 ± 2.4 years old (3.8–18.2 years) in the retained implant group and 12.3 ± 3.9 years old (5.2–21.0 years) in the removed implant group. Two sample *t* test determined that these values were not significantly different (*p* = 0.48). Mean follow-up from definitive procedure to latest follow-up was 4.1 ± 2.5 years (0.3–18.6 years) in patients with removed implants and 4.0 ± 2.3 years (0.3–9.1 years) in patients with removed implants. Two sample *t *test also found that these values were not significantly different (*p* = 0.98).

One hundred twenty-nine (55.4%) of patients in the retained implant group and nineteen (63.3%) of patients in the removed implant group had some comorbidity, and Fisher’s exact test determined that the differences in these rates were not significantly different (*p* = 1.00). In the retained cohort, 85 (36.5%) had congenital scoliosis, 60 (25.8%) had neuromuscular scoliosis, 38 (16.3%) had syndromic scoliosis, and 20 (8.6%) had idiopathic scoliosis. In the removed cohort, there were 14 (46.7%) patients with congenital scoliosis, 11 (36.7%) with neuromuscular scoliosis, 5 (16.7%) with syndromic scoliosis, and none with idiopathic scoliosis. Fisher’s exact test determined that the distribution of etiologies was not significantly different between cohorts (*p* = 0.30). The mean pre-index coronal curvature was 74.6 ± 22.0 degrees (24–138 degrees) in the retained group and 70.5 ± 24.7 degrees (32–122 degrees) in the removed group, and two-sample *t *test determined that these were not significantly different (*p* = 0.47).

The 30 patients who ultimately had their implants removed experienced a total of 0 UPRORs following removal of implants. The removed group experienced ten (33.3%) complications among five (16.7%) unique patients. Only one (3.3%) of these complications was wound related. A list of these complications is included in Supplementary Table S1. The 203 patients who retained their implants experienced a total of 10 (4.9%) UPRORs and 41 (20.2%) had complications following final lengthening. There were 9 unique retained patients (28.9%) who experienced an UPROR and 26 (12.8%) unique patients with a complication. Of these, six (2.3%) complications were wound related. Two complications (1.0%) were device or procedure related, while two (1.0%) more were listed as uncertainly related. Of the UPRORs, five (2.5%) were wound related. Again, two (1.0%) were device related or procedure related, and two (1.0%) more were listed as uncertainly related to device or procedure. A list of these complications is included in Supplementary Table S2.

Data regarding loss of curve correction were available for 20 patients in the removed implant group and 102 patients in the retained implant group. In the cohort who retained their implants, mean progression of major Cobb angle at their post-definitive procedure and at their most recent follow-up was 19.0 ± 4.3 degrees (0–80 degrees) for those with implants retained compared to 14.3 ± 12.0 degrees (1–38 degrees) for the cohort whose implants were removed (Table 2). Shapiro–Wilk test determined that both samples were not normally distributed, and Mann–Whitney U test found that these means were not significantly different (*p* = 0.18). Coronal curvature at patients’ definitive procedure was not significantly associated with progression in the retained implant group (coefficient = 0.04, *p* = 0.56) nor the removed implant group (coefficient = 0.09, *p* = 0.46) 11/20 removed patients (55.0%) and 37/101 retained patients (36.7%) had 10° or less of progression.

Data regarding changes in kyphosis were available for 10 patients in the removed implants group and 116 patients in the retained implant group. Between their post-definitive procedure and most recent follow-up, the removed implants group had a mean change in kyphosis of 15.8 ± 8.2 degrees (7–33 degrees) compared to 12.5 ± 11.1 degrees (0–68 degrees) in the retained group (Table 2). Shapiro–Wilk test determined that both samples were not normally distributed, and Mann–Whitney U test found that these means were not significantly different (*p* = 0.09). The mean time between the post-definitive X-ray date and most recent X-ray date was 3.8 years for removed implant patients and 3.7 years for retained implant patients. Mean values for pre-index measurements in both cohorts can be found in Table 1. We found no significant differences between pre-index major Cobb angle, minor Cobb angle, kyphosis, nor T1-S1 height.

**Table 1:** Comparison of pre-index measurements for those with implants retained and removed

Pre-index measurement	Implants	Mean	Comparison test (*t* test/Mann–Whitney U test)
Major Cobb angle	Retained	74.3°	*p* = 0.51
	Removed	70.5°	
Minor Cobb angle	Retained	42.2°	*p* = 0.82
	Removed	44.3°	
Kyphosis	Retained	50.3°	*p* = 0.29
	Removed	58.3°	
T1-S1 height	Retained	25.5 cm	*p* = 0.46
	Removed	25.1 cm	

**Fig. 2:**
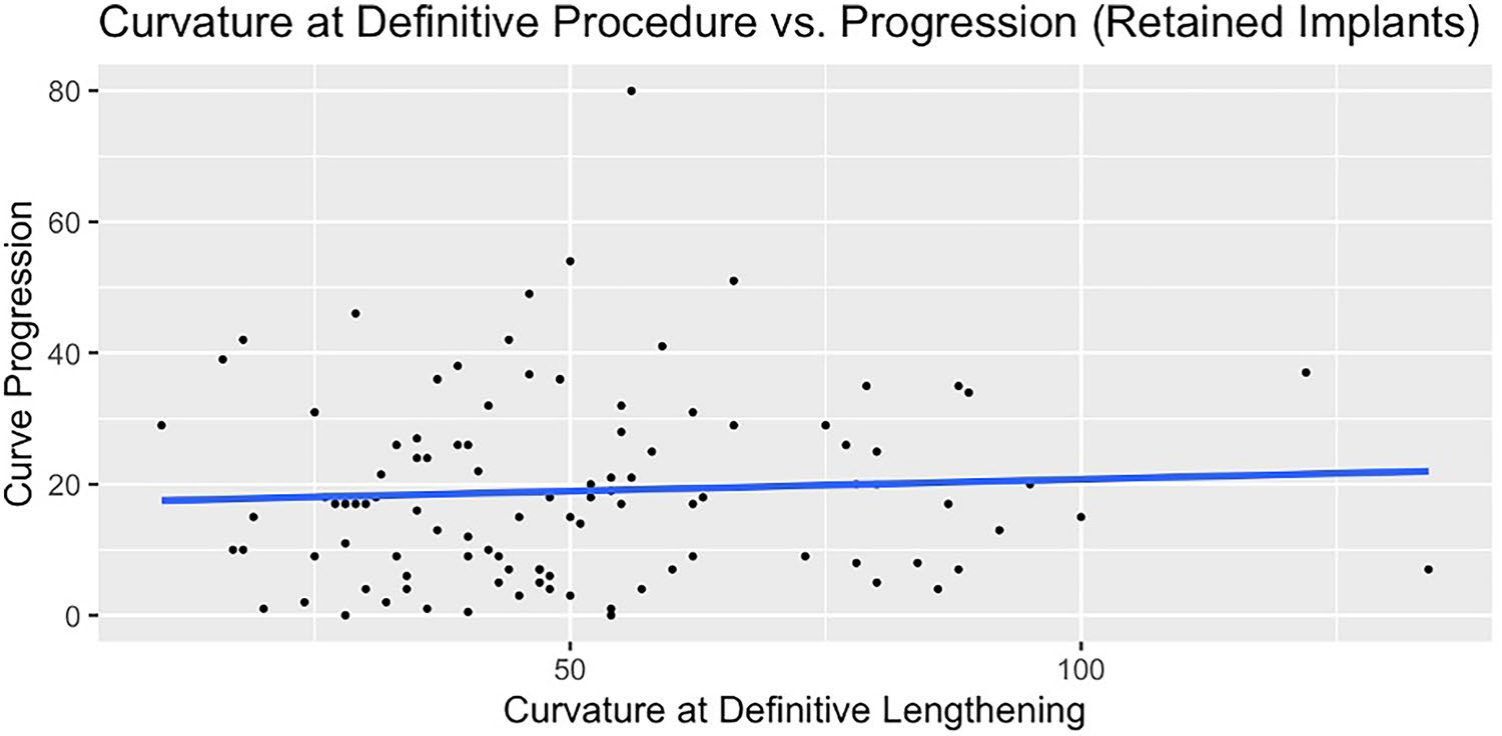
Kaplan–Meier curves modeling time to first complication following definitive procedures for patients with retained implants (above) and removed implants (below)

Kaplan–Meier survival analysis determined that patients with removed implants generally had a greater likelihood of experienced complications within approximately 2 years following definitive procedures, but retained patients were at greater risk at longer term follow-up (Fig. 2).

## Discussion

Many patients and families look forward to the “end” of their EOS journey when their planned interventions are complete and there is no anticipated return to the operating room. Typically, this “end” is marked by definitive spinal fusion. Poe-Kochert et al. [6], however, demonstrated that 20% of final fusion surgeries are, in fact, not the last procedure for these patients. It is also likely that this figure is an underestimate as Poe-Kochert et al. [6] found that some patients underwent further procedures after the conclusion of the study. If those patients who undergo a “definitive” fusion face at least a 20% reoperation rate, what happens to patients who instead choose to forego definitive fusion surgery? (Table 2)

**Table 2:** Changes in post-definitive measurements

Post-index measurement	Implants	Patients with available data	Mean change (°)	Range (°)
Major Cobb angle	Retained	102	19.0 ± 4.3	(0, 80)
	Removed	20	14.3 ± 12.0	(1, 38)
Kyphosis	Retained	116	15.8 ± 8.2	(7, 33)
	Removed	10	12.5 ± 11.1	(0, 68)

A 2016 study from the Growing Spine Study Group attempted to answer this question, comparing a cohort of patients who avoided final surgical fusion at skeletal maturity to those who underwent a final fusion procedure. Of the 167 patients included, all had TGR, and 30 patients comprised the observation group. Of these 30, 4 had implants removed. They noted no incidences of rod fracture after final distraction, although there was a unilateral separation of the rod from the connector in 1 patient. They also found no significant difference in final curve magnitude between the observation and final surgical fusion groups [8].

Our results suggest that, for patients who elect to not undergo definitive fusion, rates of complications and returns to the operating room are not insignificant prior to their last planned procedure. For patients whose definitive procedure was a final open lengthening of TGR or VEPTR, or the retained group, less than 5% experienced UPROR, but over 10% experienced complications. Moreover, no patients whose definitive procedure was implant removal, or the removed group, experienced UPROR, but one third experienced complications. While there exists limited data regarding UPROR and complications in patients who choose to remove their implants without undergoing final fusion, the rates we found in both groups are generally similar or favorable to those found in past studies analyzing growing constructs. In a study comparing rates of UPROR among multiple instrumentation strategies, Anari et al. [10] found that, within 2-year follow-up, 55 of 258 VETPR patients (21%) and 5/43 TGR patients (12%) experienced UPROR. El-Hawary et al. [11] determined that, of 63 EOS patients without rib abnormalities, 31 VEPTR patients (49%) experienced at least one complication at 2-year follow-up. Bess et al. [12] had 58% of 140 TGR patients experience at least one complication at mean 5-year follow-up.

Notably, few UPRORs and complications were device or procedure related. Only one patient in the removed implant group and two patients in the retained implant group experienced a definitively device- or procedure-related complication, and there were only two such UPRORs in the retained group. Again, these results are favorable when compared to past studies. Anari et al. [10] found that anchor failure accounted for 59% of UPRORs when considering all forms of instrumentation. In a study of 51 patients with a variety of growing constructs, Basu et al. [13] found that 53 of 82 unplanned procedures (64.6%) were implant revisions. However, these differences in rates of implant or device complications could be due to the greater variety of instrumentation methods included in these studies. Moreover, the UPRORs described by Anari et al. were more acute than in our sample as all instances were recorded within 2 years of implantation.

Previous studies have had mixed results on the maintenance of curve correction following removal of implants, and there is little data on those who had no final fusion prior to implant removal. In a study of 26 patients in which 10 had their growing rods removed, Kocyigit et al. hypothesized that autofusion would provide enough stability for these patients to avoid autofusion. However, they eventually found that nine of these ten patients experienced clinically important worsening of their deformity and required reimplantation with fusion [9]. Shen et al. [14] also agree that the results found after removal of growing rod instrumentation without final fusion should push surgeons toward final fusion for the vast majority of patients. However, Jain et al. [8] found that, in four patients who removed growing rods due to infection and did not undergo definitive fusion, two had only mild curve progression.

Certainly, there is an expectation that removing implants without a spinal fusion would result in a significant loss of curve correction. That was not necessarily the experience for all children in this group. The mean progression of coronal curvature in this cohort was approximately 14 degrees over mean 3.8 years of follow-up. Within this group, these values exhibited a wide range of 1–38 degrees. Specifically, 11/20 patients (55%) experienced less than 10 degrees or less of curve progression at mean 3.8-year follow-up. There was also no significant relationship between spinal curvature immediately following implant removal and curve progression at latest follow-up. These figures are generally much more encouraging than previously described.

**Fig. 3:**
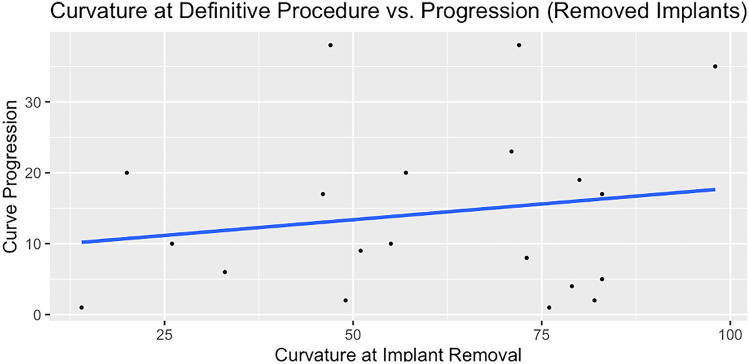
Chart demonstrating changes in curvature are correlated with curve magnitude at the time of implant removal

**Fig. 4:**
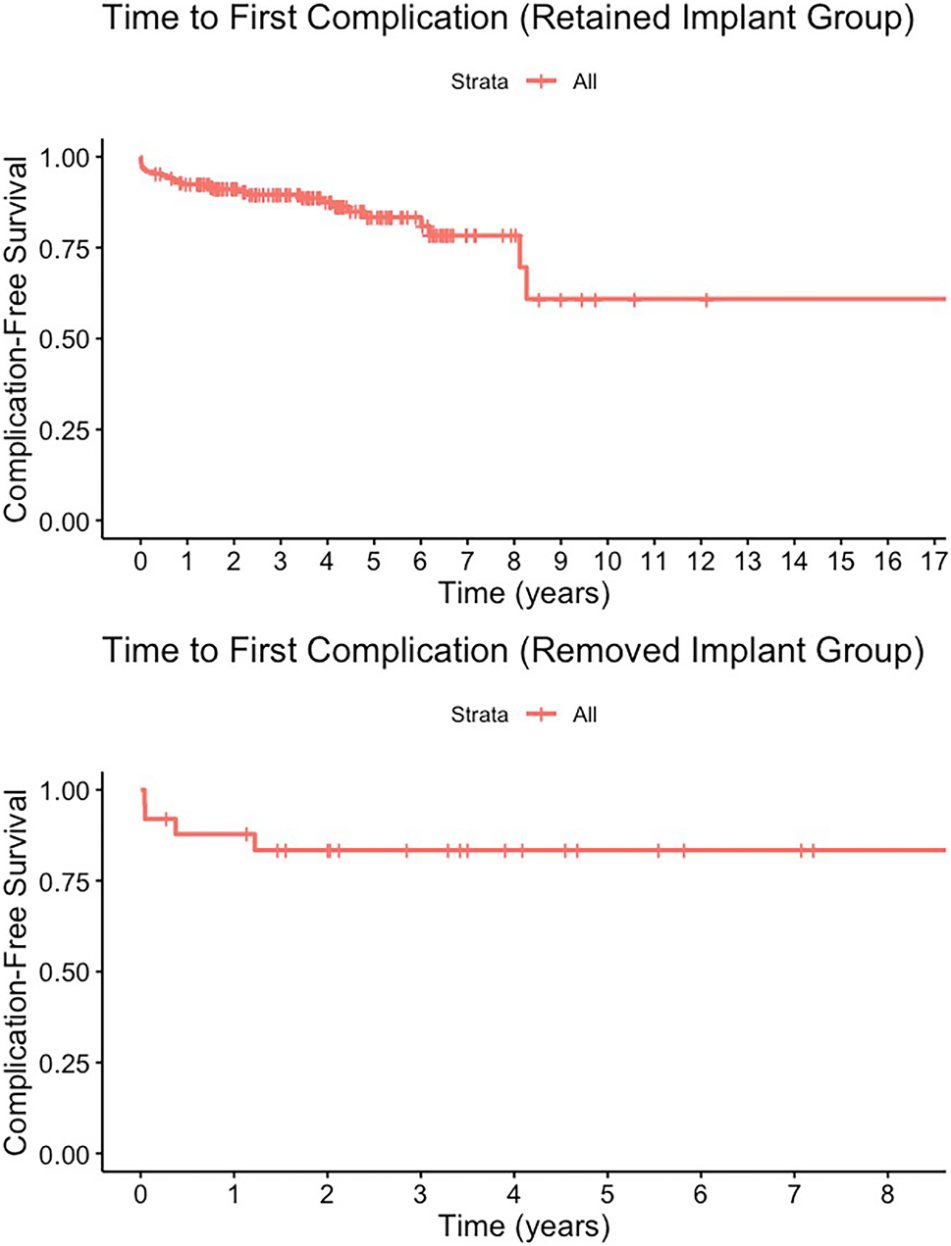
The time to first complication is demonstrated for each of the two cohorts

Our study offers a comparison between two similar cohorts of patients with removed implants and patients who did not undergo definitive fusion but also did not remove their growth-friendly instrumentation. This group also experienced moderate coronal curve loss, a mean of approximately 19 degrees. This range or curve loss was even wider than the previous cohort, exhibiting values from 0 to 80 degrees. The median curve progression was 17 degrees, and 37/101 patients (36.7%) had their curves progress 10 degrees or less at mean 3.7 year follow-up. Again, we found no significant relationship between coronal curvature following definitive lengthening and curve progression at latest follow-up. Patients with retained implants demonstrated a significantly lower rate of UPROR after undergoing their definitive final procedure than those who ultimately had removal of their implants as demonstrated in Fig. 2. While these patterns may not translate across all diagnoses, curve patterns, or magnitudes, retaining TGR or VEPTR implantations may offer a path to correction without undergoing the risks of definitive fusion (Figs. 3, 4).

Limitations of this study include the retrospective nature of the review, the potential for data error when using a multi-center database, the potential bias of surgeons’ decisions to retain or remove implants, and the exclusion of implant types other than TGR or VEPTR. While this data offers valuable information regarding rates of UPROR and complications following definitive procedures, a considerable number of patients in both cohorts did not have available data regarding post-definitive coronal curvature and sagittal kyphosis measurements, making it difficult to draw similar conclusions regarding coronal curve progression. Despite these results, one might also expect that those with removed implants would experience greater coronal curve and sagittal kyphosis progression. The lack of significant difference in post-operative progression could be attributable to spontaneous autofusion in the removed cohort, but autofusion was not necessarily recorded in all removed implant patients. Finally, in the context of EOS, a minimum follow-up of 2 years from index is still very limited when considering the long-term outcomes of these patients.

Future studies should evaluate outcomes for patients who undergo treatment with other implants or surgical techniques including Shilla, magnetically controlled growing rods, and others, who choose to forego definitive fusions. This group of patients could then be revisited at a later time point to see if the survivorship of their implants is maintained or if any more complications emerge. Longer follow-up time would also allow us to determine whether curve progression begins to worsen in through adolescence or after growth has stopped.

In conclusion, this study demonstrates that growth-friendly graduates who are observed but do not undergo a final fusion exhibit low rates of UPROR and complications following their definitive procedure, regardless of whether their implants were retained or removed. Contrary to expectation, the curve magnitude is well maintained in most members in this cohort whether implants were removed or kept. While the risks typically associated with TGR and VEPTR are still present, patients and surgeons may consider maintaining growth-friendly implantations as an alternative to the risks associated with definitive spinal fusion at a young age.

## Supplementary Information

Below is the link to the electronic supplementary material.

**Supplementary Table 1**. List of Complications for Patients with Removed Implants (PNG 64 kb)

**Supplementary Table 2**. List of Complications for Patients with Retained Implants (PNG 115 kb)
