# Supplementary figures and images for: Leave it alone: the natural history of growth-friendly graduates without a final fusion
Source: Spine Deform. 2026 Jan 24;14(3):953–9. doi: 10.1007/s43390-025-01187-9 (PMC13282303; doi:10.1007/s43390-025-01187-9)

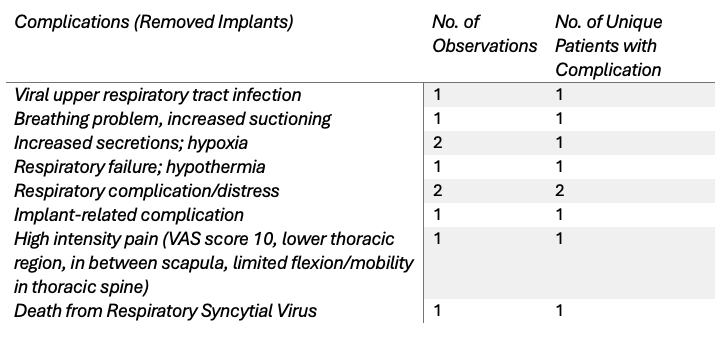

Supplement: Supplementary file 1 — Supplementary Table 1. List of Complications for Patients with Removed Implants (PNG 64 kb) [file 43390_2025_1187_MOESM1_ESM.png]

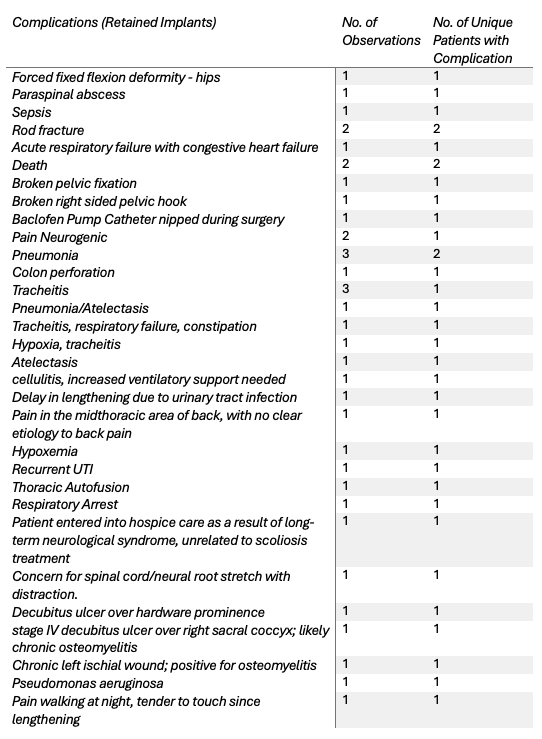

Supplement: Supplementary file 2 — Supplementary Table 2. List of Complications for Patients with Retained Implants (PNG 115 kb) [file 43390_2025_1187_MOESM2_ESM.png]
